# Tetris-Style Stacking Process to Tailor the Orientation of Carbon Fiber Scaffolds for Efficient Heat Dissipation

**DOI:** 10.1007/s40820-023-01119-0

**Published:** 2023-06-07

**Authors:** Shida Han, Yuan Ji, Qi Zhang, Hong Wu, Shaoyun Guo, Jianhui Qiu, Fengshun Zhang

**Affiliations:** 1 The State Key Laboratory of Polymer Materials Engineering, Sichuan Provincial Engineering Laboratory of Plastic/Rubber Complex Processing Technology, Polymer Research Institute of Sichuan University, Chengdu, 610065 People’s Republic of China; 2 Department of Mechanical Engineering, Faculty of Systems Science and Technology, Akita Prefectural University, 015‐0055, Akita, Japan; 3 Institute of Chemical Materials, China Academy of Engineering Physics, Mianyang, 621900 People’s Republic of China

**Keywords:** Carbon fiber, Magnetic field, Thermal management, Thermally conductive composites

## Abstract

As the miniaturization of electronic devices and complication of electronic packaging, there are growing demands for thermal interfacial materials with enhanced thermal conductivity and the capability to direct the heat toward heat sink for highly efficient heat dissipation. Pitch-based carbon fiber (CF) with ultrahigh axial thermal conductivity and aspect ratios exhibits great potential for developing thermally conductive composites as TIMs. However, it is still hard to fabricate composites with aligned carbon fiber in a general approach to fully utilize its excellent axial thermal conductivity in specific direction. Here, three types of CF scaffolds with different oriented structure were developed via magnetic field-assisted Tetris-style stacking and carbonization process. By regulating the magnetic field direction and initial stacking density, the self-supporting CF scaffolds with horizontally aligned (HCS), diagonally aligned and vertically aligned (VCS) fibers were constructed. After embedding the polydimethylsiloxane (PDMS), the three composites exhibited unique heat transfer properties, and the HCS/PDMS and VCS/PDMS composites presented a high thermal conductivity of 42.18 and 45.01 W m^−1^ K^−1^ in fiber alignment direction, respectively, which were about 209 and 224 times higher than that of PDMS. The excellent thermal conductivity is mainly ascribed that the oriented CF scaffolds construct effective phonon transport pathway in the matrix. In addition, fishbone-shaped CF scaffold was also produced by multiple stacking and carbonization process, and the prepared composites exhibited a controlled heat transfer path, which can allow more versatility in the design of thermal management system.

**Supplementary Information:**

The online version contains supplementary material available at 10.1007/s40820-023-01119-0.

## Highlights

- Carbon fiber (CF) scaffolds with horizontally aligned, diagonally aligned and vertically aligned structure were fabricated via magnetic field-assisted Tetris-style stacking and carbonization process.
- The obtained CF scaffolds/ polydimethylsiloxane composites showed ultrahigh thermal conductivity (above 40 W m^−1^ K^−1^) in the fiber alignment direction.
- Fibers with different alignment direction can be combined by multiple stacking and carbonization process, allowing for the efficient heat transfer along customized paths.

**Supplementary Information:**

The online version contains supplementary material available at 10.1007/s40820-023-01119-0.

## Graphical abstract

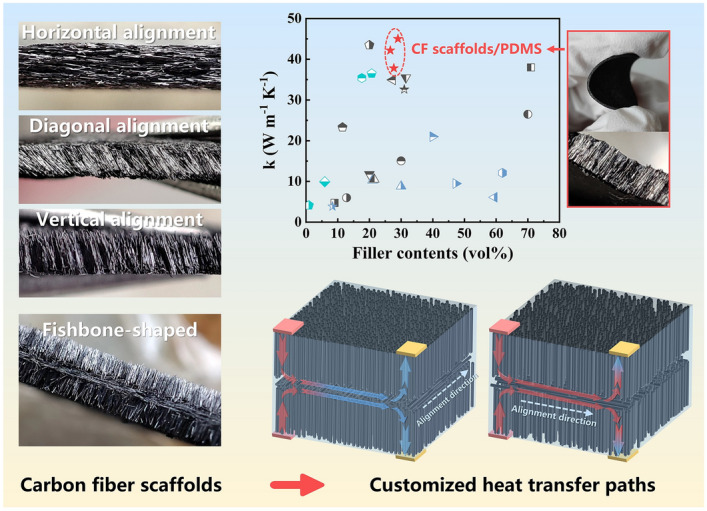

## Introduction

With the development of electronic devices toward miniaturization, integration and high-power consumption, highly efficient heat dissipation has drawn increasing attention in the past few years to diminish the heat accumulation during device operation [1, 2]. Consequently, thermal management systems are ingeniously designed into electronic devices to facilitate the heat transfer to the outside and avoid working under overheating condition. As an important part of thermal management system, thermal interfacial materials (TIMs) are used for filling the gaps between metal-plate heat sink and heat generator to reduce thermal contact resistance and solve the interfacial heat transfer problem [3–5]. However, the thermal conductivity of conventional TIMs made by traditional blending process is hard to exceed 10 W m^−1^ K^−1^ even at the filler content greater than 60 wt%. The poor enhancing efficiency will inevitably increase the density and the compress modulus of TIMs, which is harmful to the performance of TIMs in practical application [6–10]. Therefore, TIMs with enhanced thermal conductivity and the capability to direct the heat toward heat sink for highly efficient heat dissipation are urgently required, due to the dramatic increase in heat flux and the miniaturization of electronic devices. Furthermore, facing with the complication of electronic packaging, electronic components become more closely arranged and are vulnerable to the thermal interference from adjacent components. The limited space of the device may make the design of thermal management system beyond the traditional configuration of vertical heat transfer, which causes the need for TIMs with tunable heat transfer direction to optimize the cooling structure [11, 12].

According to the phonon heat transfer mechanism, the thermal conductivity of TIMs can be generally improved by incorporating fillers with high intrinsic thermal conductivity such as carbon nanotube (CNT) [13–17], graphite [18–20] and graphene nanoplatelet (GNP) [21–24], to construct effective phonon transport pathways in the matrix. Beside the above fillers, Pitch-based carbon fiber (CF), as one of the best candidates for TIMs, has not only ultra-high thermal conductivity in the axial direction (reach 1200 W m^−1^ K^−1^), but also high aspect ratios and sub-micron length, which allow CF to be more easily aligned and interconnected to form continuous heat transfer paths with reduced filler-filler interfaces in heat transfer direction. Due to the anisotropic properties of CF, lots of strategies have been developed to construct orientation structure by applying electric field [25–27], magnetic field [28–31], flow field [32–34] and templates [35–40] for making full use of its axial thermal conductivity. For example, Kenji et al. [27] prepared a vertically aligned CF structure via electrostatic flocking, and the obtained TIMs showed high through-plane thermal conductivity of 23.3 W m^−1^ K^−1^ with a filler loading of 13.2 wt%. Zhang et al. [41] constructed vertically aligned CF in PDMS using specially designed squeezing mold. The thermal conductivity of the composites reached 38 W m^−1^ K^−1^ with the loading of 24 vol% CF and 47 vol% Al_2_O_3_. Lin et al. [36] fabricated oriented CF/PDMS composites by pre-arranging the fibers before impregnating the matrix. The through-plane thermal conductivity of the composites was 34.94 W m^−1^ K^−1^ at the fiber content of 44.46 wt%. In particular, because of the anisotropic magnetic susceptibility [42, 43], CF can be oriented in magnetic field without the modification of magnetic particles, showing attractive features with simplicity, safety, and high efficiency. Miao et al. [31] used superconducting magnet to orient CF in PDMS at a magnetic field strength of 9 T. The aligned CF in the thickness direction resulted in high through-plane thermal conductivity of 26.49 W m^−1^ K^−1^. However, in this case, very high magnetic field strength is necessary to generate sufficient magnetic torque for CF to overcome the resistance of hydrodynamic torque exerted by surrounding viscous medium. Thus, limited by special mold and equipment, it is still hard to construct aligned structure of CF in the sheet by a general method to fully utilize its excellent axial thermal conductivity for application as TIMs.

As the miniaturization of electronic devices and the complexity of electronic packaging make the design of cooling structure more difficult, the need for high thermal conductivity of TIMs is not limited to the thickness direction. TIMs with tunable thermally conductive direction are able to regulate the heat transfer paths, which allows more versatility in the design of thermal management system [12]. Although most of strategies can achieve the orientation of fillers in varied direction, the control of filler orientation and the combination of fillers with different orientation direction for regulating heat transfer paths are rarely studied. Therefore, it is a challenge to develop TIMs with both high thermal conductivity and controlled heat transfer paths to cope with the rapidly evolving electronic devices.

In this work, we proposed a route for producing novel CF scaffolds with oriented and tightly stacked structure via commercial permanent magnets-assisted Tetris-style stacking and carbonization process. By regulating the magnetic field direction and initial stacking density, three self-supporting CF scaffolds with horizontally aligned (HCS), diagonally aligned (DCS) and vertically aligned (VCS) fibers were fabricated. After embedding polydimethylsiloxane (PDMS), the resultant HCS/PDMS and VCS/PDMS composites exhibited ultrahigh thermal conductivity of 42.18 and 45.01 W m^−1^ K^−1^ in the fiber alignment direction, separately, showing excellent directional heat transfer capability resulted from highly ordered and continuous CF skeleton. Furthermore, fishbone-shaped CF scaffold (FCS) was also prepared by this method, and the obtained FCS/PDMS can direct heat toward the copper plate in the same plane as the heat source. Our strategy provides insight into CF-based thermal pad, which could diversify the design of thermal management system.

## Experiment

### Materials

Pitch-based CF (XN-100-25 M) with axial thermal conductivity of 900 W m^−1^ K^−1^ was obtained from Nippon Graphite Fiber Corporation, and the average length and diameter are 250 and 10 μm (Fig. S1), respectively. PDMS (Sylgard 184) was purchased from Dow Corning Co., Ltd. Resole phenolic resin (FB resin, Boron-modified) was obtained from Tianyu High-Temperature Resin Materials Co., Ltd. Ethanol and Ethyl acetate were supplied by Chengdu Chron Chemical Co., Ltd. All chemicals were of analytical reagent grade and used directly.

### Preparation of CF Scaffolds

**Fig. 1:**
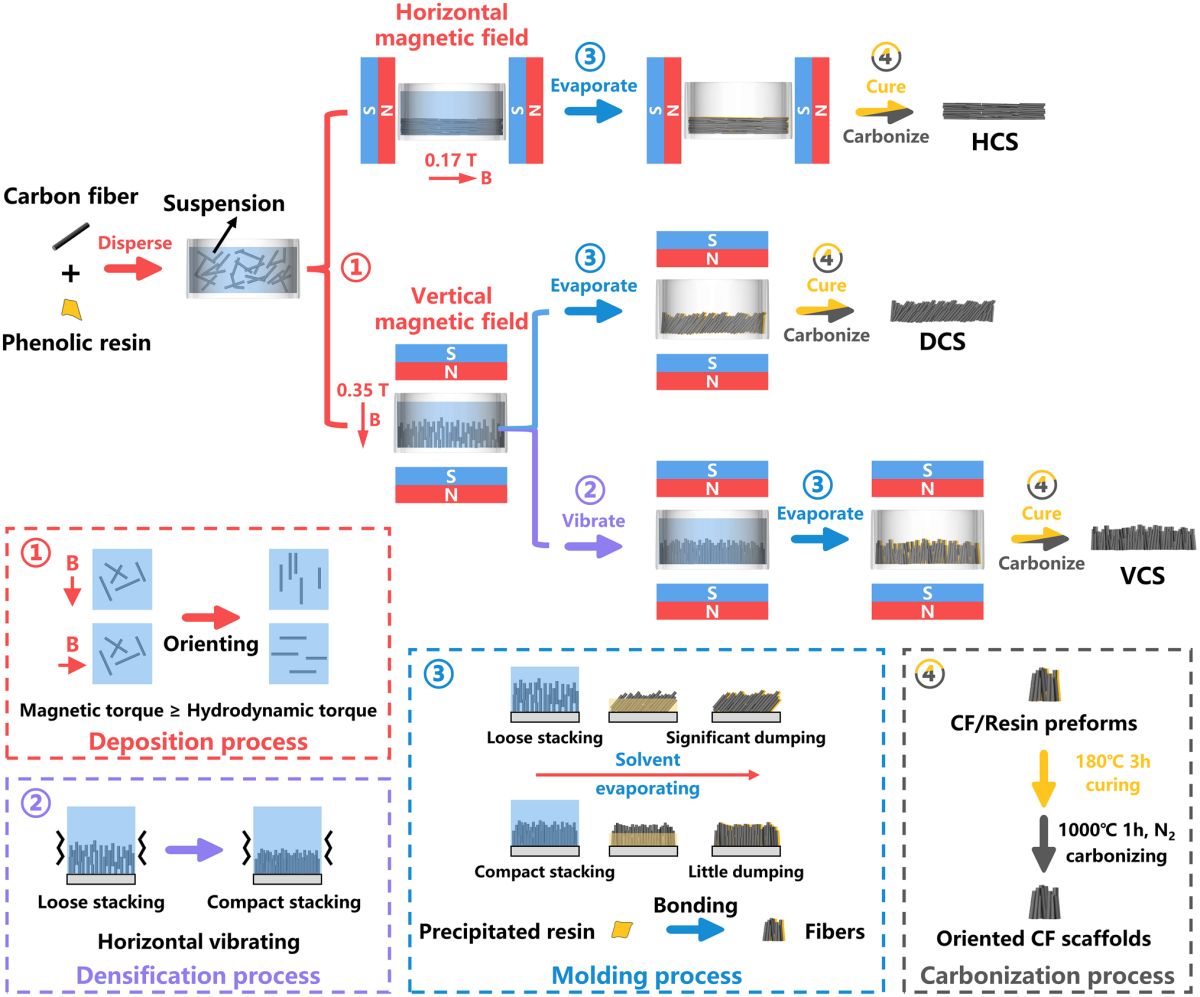
Schematic illustrating the fabrication process of the oriented CF scaffolds with the corresponding structural change for each step

The schematic of preparation process can be seen in Fig. 1. Certain amount of resole phenolic resins was dissolved in 5 g ethanol to form resin solution under stirring for 30 min. CF with different contents was then added to the solution and stirred for 15 min to disperse them well. The concentration of CF in resin solution was varied from 5 to 10 wt%, and the mass ratios of CF and resole phenolic resin were 10:2 constantly. After mixing, the suspension was poured into a polystyrene dish (diameter: 33 mm) forming a liquid height of about 10 mm and immediately moved to the magnetic field generated by two commercially available NdFeB magnets (length × width × thickness: 46 mm × 46 mm × 22 mm). Due to the anisotropic susceptibility, CF was aligned along the magnetic field during deposition process and land horizontally or vertically on the bottom of the dish. Especially for VCS, once all CFs have settled to the bottom, the magnets and dish were put on a vibrating platform and exerted horizontal vibration with frequency of 25 Hz and amplitude of 0.5 mm for 1 min to make fibers tightly packed.

Subsequently, the mixture was dried at room temperature for 48 h in the magnetic field to remove the solvent, during which the resins will precipitate and bond the closely packed fibers together. Finally, the shaped CF/resin preforms were cured at 180 °C for 3 h, followed by the carbonization at 1000 °C in nitrogen atmosphere for 1 h to obtain a series of CF scaffolds. The HCS-*x*, DCS-*x* and VCS-*x* were used to name prepared scaffolds with different contents of CF, where *x* is the concentration of CF in solution. For example, VCS-7 was prepared by using the suspension with CF concentration of 7 wt%. The CF scaffold without magnetic field effect were also made to compare, which was named as CS-*x*.

Especially, FCS was prepared by multiple stacking and carbonization process with different magnetic field direction (Fig. S2). First, horizontal magnetic field was adopted, and the HCS/resin preform was produced and cured at 180 °C for 3 h using the same way as above. Then, the cured HCS/resin was placed on the bottom of vessel and VCS/resin preform was formed on its surface under the vertical magnetic field. To prevent ethanol from dissolving the resin and destroying the orientation structure, the VCS/HCS/resin preform was cured again and VCS/resin preform was produced on the other surface following the same step. After curing and carbonization process, FCS with a three-layer structure was finally prepared.

### Preparation of CF Scaffolds/PDMS Composites

The CS/PDMS, HCS/PDMS, DCS/PDMS, VCS/PDMS and FCS/PDMS composites were fabricated by the infiltration of PDMS into permeable CF scaffolds with the assistance of vacuum. Initially, PDMS prepolymer, curing agent and ethyl acetate were mixed together with the weight ratio of 30:1:3. Then, the mixture was injected into the prepared CF scaffolds and place the samples in vacuum oven for more than 4 h to remove their air bubbles at room temperature. Finally, the samples were cured at 80 °C for 6 h and polished to obtain CF scaffolds/PDMS composites.

### Characterization

Various methods were introduced to investigate the micromorphology of CF scaffolds and composites. Scanning electron microscope (SEM, JEOL JSM-5900LV, Japan) was used to observe the microstructure of CF, CF scaffolds and CF scaffolds/PDMS composites. Microcomputed tomography (Micro-CT, ZEISS Xradia 520 Versa, Germany) was used to reconstruct the 3D microstructure of CF scaffolds at a resolution of 2 μm. X-ray diffraction (XRD) and Raman spectroscopy were used to determine the integrity of crystal lattices in carbon fibers. XRD patterns were obtained by diffractometer (Rigaku Ultima IV, Japan) using a Cu Kα radiation (*λ* = 0.15406 nm) at 40 kV and 100 mV, 2*θ* from 10° to 90°. Raman spectroscopy was provided by micro-Raman spectrometer (Renishaw inVia Reflex, UK) with He–Ne laser excited at 532 nm. Thermal diffusivity (α) of composites was measured by laser flash apparatus (LFA, Netzsch LFA 467, Germany) in the nitrogen atmosphere from 25 to 100 °C. Specific heat capacity (*C*_p_) was obtained by differential scanning calorimetry (DSC, TA Q20, USA) with a heating rate of 20 °C min^−1^ from − 5 to 130 °C under nitrogen. The thermal conductivity (*k*) can be calculated by the equation: *k* = *α* × *C*_p_ × ρ, where ρ is the density of sample determined by the water displacement method. Infrared images were captured by using an infrared camera (FLIR T620, USA). Thermogravimetric analysis (TGA) of CF, PDMS and CF scaffolds/PDMS composites was carried out from 30 to 800 °C with a heating rate of 10 °C min^−1^ under nitrogen atmosphere to calculate the filler content in composites. The apparent viscosity of resin/ethanol solutions with different concentrations was measured by a rotational rheometer (Anton Paar MCR302, Austria) in the shear rate range of 1 to 100 s^−1^.

## Results and Discussion

### Preparation and Structural Characterization of CF Scaffolds

Figure 1 schematically shows our strategy for the development of CF scaffolds, with the corresponding structural change for each step of fabrication process. In the first procedure, well-dispersed suspensions, consisting of CF and phenolic resin/ethanol solution, were immediately transferred to a parallel magnetic field generated by two NdFeB magnets. Due to the difference of magnetic susceptibility in axial and radical directions, CF will align along the magnetic field direction when the generated magnetic torque larger than the hydrodynamic torque exerted by surrounding solution. According to the formula (1) [44, 45],

1 $$\tau = \frac{{6 \cdot \mu_{0} \cdot \eta }}{{F\left( D \right) \cdot \chi_{a} \cdot B^{2} }}$$

the alignment relaxation time (*τ*) is a function of viscosity (*η*), vacuum magnetic permeability (*μ*_0_), aspect ratios (*D*), anisotropic magnetic susceptibility (*χ*_a_) and magnetic field strength (*B*). Once the *τ* is smaller than the deposition time (*t*), CF can finish the orientation and maintain the direction to land the bottom of the dish. For predicting the *τ*, parameters (*D* = 25, *χ*_a_ = 8.9 × 10^–6^ [44]) were brought into the equation and the curve of *τ* versus *η* is shown in Fig. S3a. As viscosity increases from 1 to 5 mPa s, the τ rises from 0.6 to 2 s under the magnetic strength of about 0.3 T. To access the feasibility, macro-camera was applied to record this procedure (Video S1 and Fig. S3c). When the suspension of 10 mm height was placed on a magnet, CF completed the turning in about 2 s as indicated by yellow arrow and then deposited to the bottom in an aligned position like the I-shaped Mino in Tetris. It can be seen that the time required for orientation was in accordance with the predicted* τ* and much less than 20 s, which is the time for most of CF to complete the deposition. Thus, almost all of the CF can be oriented in the deposition process at a low magnet field strength, which create a basis for preparation of CF scaffolds with oriented structure.

Subsequently as solvent evaporating, the resins gradually precipitated on the surface of the fibers and bonded them together to fix the stacked orientation structure (Fig. S4). However, after the deposition, vertically aligned CF was loosely stacked and there were large gaps between the fibers. So, the CF will inevitably fall over by gravity and rearranged at a certain angle as solvent diminishing. To enhance the initial stacking density and avoid dumping, horizontal vibration was adopted after the deposition and the gaps between the fibers were reduced via local displacement of CF. The height of CF stacked on the bottom was decreased clearly after the vibration illustrated in Fig. S5, indicating that the fibers were closely stacked and orderly aligned. As the precipitated resin bonding fibers, the preformed CF/resin scaffolds were cured and then placed between two ceramic plates with fixed thickness for high-temperature treatment at 1000 °C to carbonize the resin. During the carbonization, the samples were limited in the thickness direction to avoid warping. Consequently, controlling the magnetic field direction and initial stacking density, three CF scaffolds with HCS, diagonally aligned and vertically aligned structure were obtained.

**Fig. 2:**
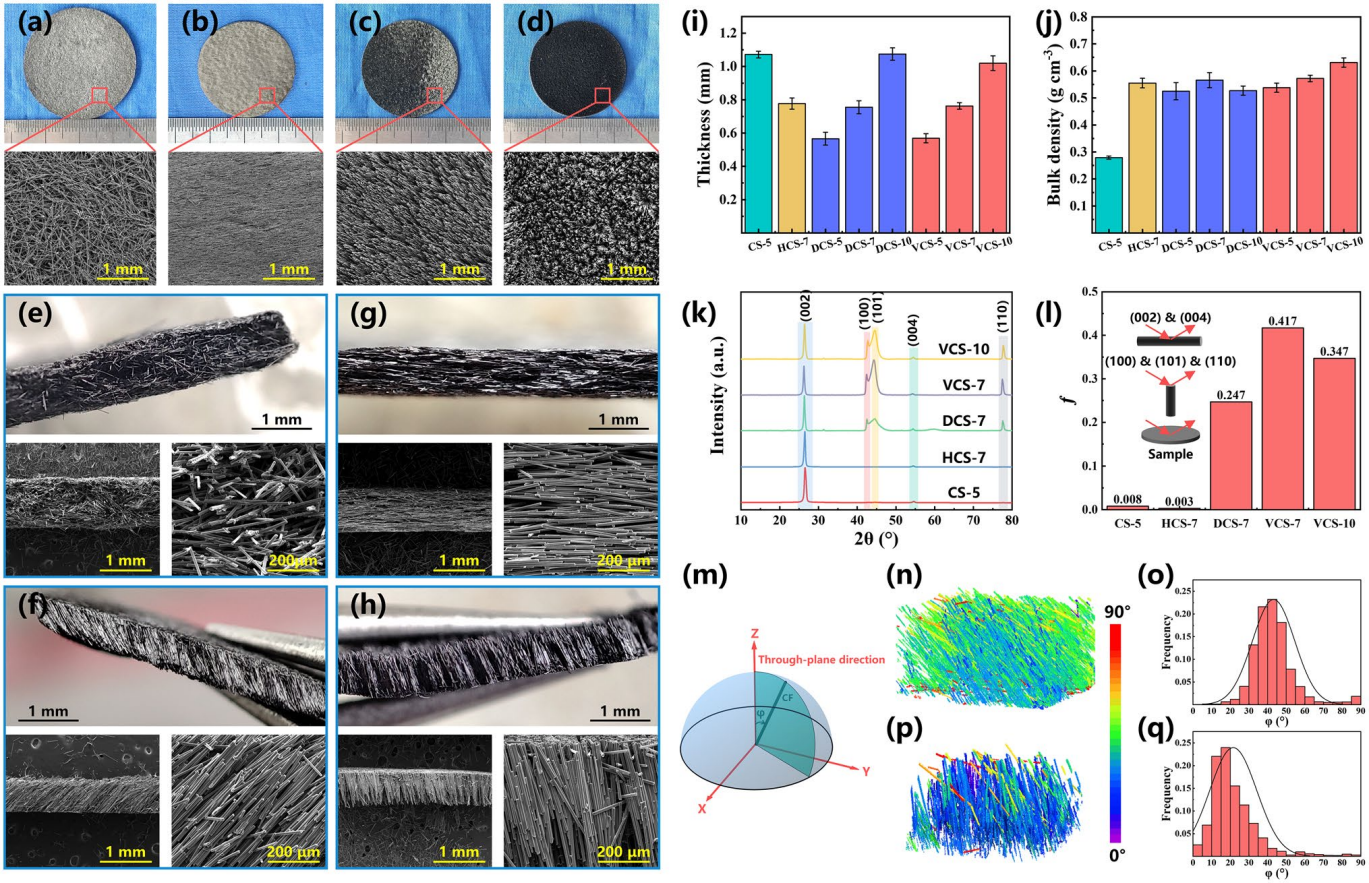
Macro-photographs and SEM images of surfaces and cross sections of prepared CF scaffolds: **a, e** SC-5, **b, g** HCS-7, **c, f** DCS-7 and **d, h** VCS-7. **i** Thickness and **j** bulk density of CF scaffolds varied with the fiber concentration in suspension during preparation. **k** XRD patterns of the surface of CF scaffolds and **l** the corresponding ratio ƒ of *I*_(100)_/(*I*_(100)_ + *I*_(002)_) in different samples. **m** Schematic of angle φ between the fiber axial and the through-plane direction. Micro-CT pictures and statistical results of *φ* in CF scaffolds: **n, o** DCS and **p, q** VCS

As mentioned above, magnetic field and vibration are two critical steps in constructing an oriented CF scaffold, which serves to orient CF and densify the deposited fibers, respectively. For comparison, the CF scaffolds without magnetic field and vibration were also prepared. The macro-photographs and SEM images of the four CF scaffolds are exhibited in Fig. 2a–h. As shown in the photographs, all CF scaffolds demonstrated good integrity and can be self-supporting after 1000 °C carbonization treatment. The CS and HCS exhibited a bright surface while the surfaces of DCS and VCS were dull, where the surface of VCS was nearly black and showed light–trapping properties (Fig. 2a-d). The distinct response to light can be ascribed to the difference in surface morphology. Morphological analysis was performed using SEM, and it can be observed that CF in HCS, DCS and VCS demonstrated remarkable alignment with different orientation angle in the presence of magnetic field, while the fibers in CS were randomly arranged after natural settlement (Figs. 2a, e and S6a, e). In HCS, as horizontally aligned along the direction of magnetic field, CF was parallel to each other and tightly piled together forming masonry-like structure (Fig. 2b, f). For DCS, vertically aligned fibers dumped by gravity during the molding process, showing oriented structure with an inclination of 40°–60° in vertical direction. But for VCS, fibers showed forest-like structure and almost vertically aligned with little inclination as illustrated in cross-sectional images (Fig. 2h). Because of compact stacking of CF after the vibration, the dumping was suppressed during solvent evaporation and the fibers can be held up by bunching with adjacent fibers to maintain vertical alignment (Fig. S6d, h). Accordingly, the light arriving at the VCS surface entered the spaces between the neighboring fibers and was absorbed after multiple reflections. Photothermal properties of the CF scaffolds also reflected the morphological difference, and it can be seen in Fig. S7 that VCS showed the fastest heating rate and the equilibrium temperature reached 90 °C at the light density of 100 mW cm^−2^, which was about 18 °C higher than that of the CS and HCS.

Additionally, the effect of fiber concentrations on CF scaffolds was also studied and the corresponding SEM images can be seen in Fig. S8. As the increment of CF concentration, the structure and bulk density of CF scaffolds showed little change while the thickness increased apparently (Fig. 2i, j). Due to the closely packing of CF, the thickness of HCS, DCS and VCS were similar at the same fiber concentrations and much smaller than that of CS, which had large space between randomly distributed fibers. This was also evidenced by the difference in bulk density and the scaffolds with tightly stacked structure showed higher bulk density. However, there were some unoriented fibers at the bottom of DCS-10. Because of the special hindrance of fiber alignment in highly concentrated suspension and compression of upper fibers after solvent evaporation, it was hard for the fibers at the bottom to maintain the oriented arrangement, which could be the reason for the formation of the morphology in DCS-10.

Considering the ultrahigh axial thermal conductivity of CF, through-plane thermal conductivity of composites was significantly influenced by orientation of fibers. The degree of orientation in the through-plane direction was qualitatively characterized by XRD due to the different diffraction patterns of CF obtained from the surface and cross section [46, 47]. As shown in Fig. 2k, i, the XRD patterns of CF scaffolds in surface scan were obtained and normalized. Sharp diffraction peaks observed at about 26.42°, 42.74°, 44.32°, 54.30° and 77.48° can be indexed to the corresponding lattice plane of (002), (100), (101), (004) and (110), respectively. There were significant difference in the diffraction patterns of the scaffolds with different fiber alignment, and the greater difference in the diffraction peaks between the two directions means the better orientation in vertical direction. The ratio* f* of *I*_(100)_/(*I*_(100)_ + *I*_(002)_) was calculated, and it can be seen that the VCS showed a higher value than that of DCS, reflecting greater fiber alignment in vertical direction. For better visualizing the fiber orientation, micro-CT was performed to quantitatively describe the alignment of CF in DCS and VCS. The angle* φ* between the fiber axial and the through-plane direction (*z* axis) was introduced to indicate the degree of orientation (Fig. 2m). The values of φ were expressed by color and the individual fibers with matching color as shown in Fig. 2n, p and o, q exhibited the distribution of *φ.* A cold color (purple) indicated smaller value of *φ*, whereas a warm color (red) indicated a larger angle. It can be observed that most of fibers in DCS were green and the value of *φ* was in the range of 30°–50°, while the fibers in VCS were mainly blue, indicating that vertical orientation of fibers was well maintained after the vibration.

### Thermal Conductivity of CF Scaffolds/PDMS Composites

**Fig. 3:**
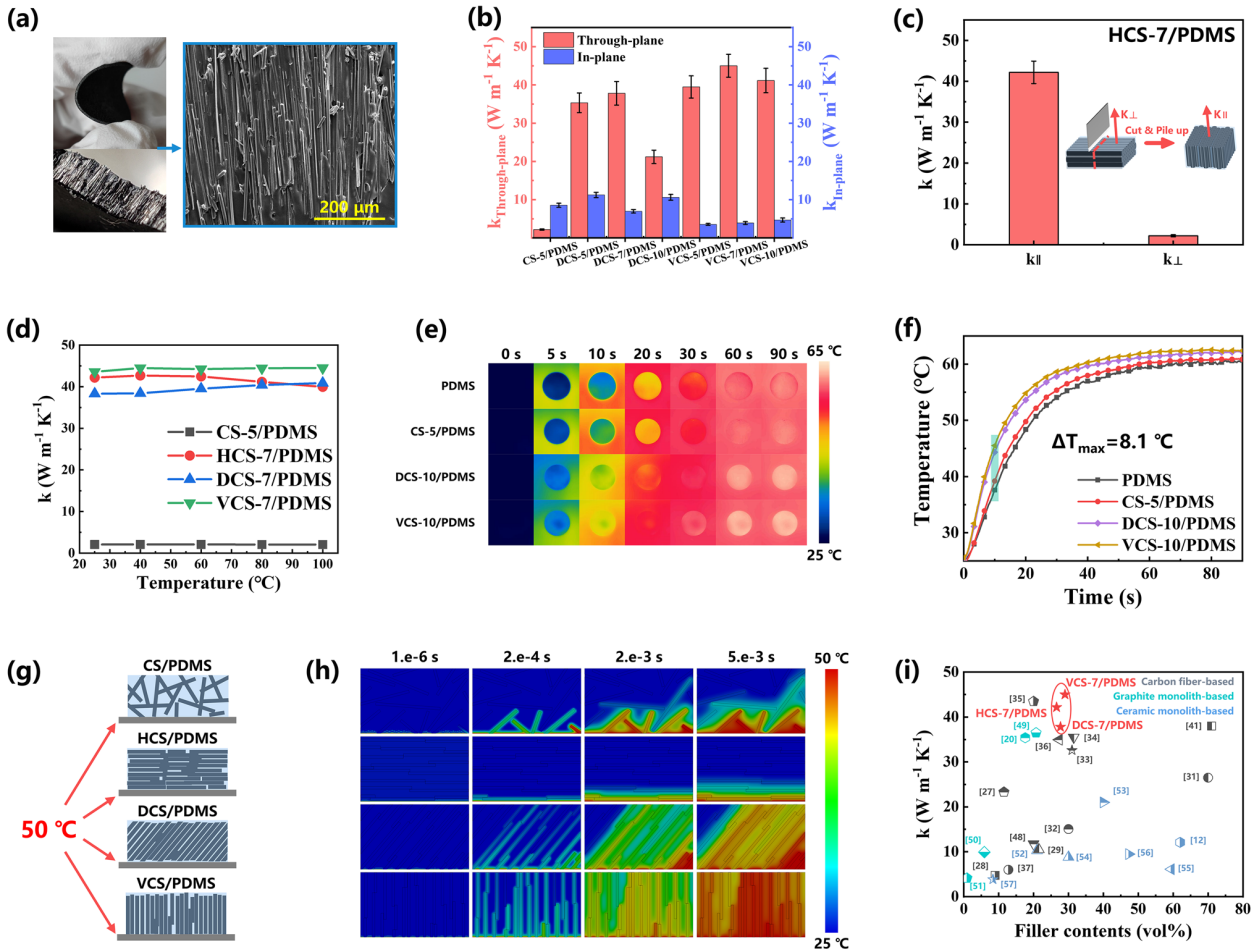
**a** Photographs and cross-sectional SEM images of VCS-7/PDMS. **b** Through-plane and in-plane thermal conductivity of CF scaffolds/PDMS. **c** Thermal conductivity of HCS-7/PDMS in the direction of fiber alignment and its vertical direction. **d** Environmental temperature-dependent thermal conductivity of CF scaffolds/PDMS. **e** Infrared thermal images and **f** the corresponding temperature curves versus heating time of PDMS, CS-5/PDMS, DCS-10 and VCS-10. **g** Schematic of the simulation models and **h** the calculated transient temperature distribution for CS/PDMS, HCS/PDMS, DCS/PDMS and VCS/PDMS. **i** Comparison of thermal conductivity of our CF scaffolds/PDMS with previously reported thermally conductive composites [12, 20, 27–29, 31–37, 41, 48–57]

Based on the interconnected and permeable structure with several orderly aligned CF stacked in the specific direction, the HCS, DCS and VCS are expected to be favorable thermal conductive fillers to develop TIMs with enhanced thermal conductivity and directional heat transfer capability for highly efficient heat dissipation in electronic packaging. Accordingly, CF scaffolds/PDMS composites were prepared by vacuum-assisted infiltration of PDMS, and the contribution of CF scaffolds with different structure on the heat transfer capability of composites was investigated. Figures 3a and S9 illustrate the macro-photographs and cross-sectional SEM images of as-prepared CF scaffolds/PDMS. It can be observed that PDMS filled in the gaps of between the fibers, and all scaffolds can keep their characteristic structure after incorporating with PDMS due to their good structural stability. Since CF scaffolds with different stacked structure showed varied bulk density, the volume fraction of CF scaffolds in composites was determined based on the TGA analysis (Fig. S10 and Table S1). The filler contents of HCS/PDMS, DCS/PDMS and VCS/PDMS were in the range of 26.6–29.7 vol%, while that of SC/PDMS was about 14.3 vol%.

The through-plane (*k*_through-plane_) and in-plane (*k*_in-plane_) thermal conductivity of composites were evaluated using the laser flash technique, and detailed parameters can be found in Table S2. Figure 3b shows the thermal conductivity of different composites, and it can be seen that PDMS filled with VCS showed relatively high thermal conductivity in the through-plane direction. The *k*_through-plane_ and *k*_in-plane_ of VCS-7/PDMS reached 45.01 and 3.9 W m^−1^ K^−1^, respectively, demonstrating high anisotropy ratio (*k*_through-plane_/*k*_in-plane_) of 11.54 due to the vertically aligned and interconnected structure of VCS. The vertically aligned fibers can form thermal transfer pathway in the thickness direction, allowing for more efficient heat transfer compared to SC-5/PDMS with random distributed fibers (2.18 W m^−1^ K^−1^). In contrast, because of the diagonal arrangement of fibers, the DCS-7/PDMS had a reduced *k*_through-plane_ and increased *k*_in-plane_ of 37.81 and 6.97 W m^−1^ K^−1^, respectively, exhibiting lower anisotropy ratio of 5.42. It is worth noting that the anisotropy ratio of DCS-10/PDMS was lowest among the DCS-filled composites, which can be ascribed to the existence of unoriented fibers at the bottom of DCS-10. Especially, due to the unidirectional arrangement of fibers in the plane, HCS-7/PDMS was cut into 2 mm wide sheets along the direction perpendicular to fiber orientation and stacked in the holder to determine the thermal conductivity (*k*_∥_) in fiber alignment direction. As illustrated in Fig. 3c, HCS-7/PDMS showed excellent thermal properties along fiber alignment direction, yielding a *k*_∥_ of 42.18 W m^−1^ K^−1^, which was comparable to the *k*_through-plane_ of VCS-7/PDMS. Furthermore, environmental temperature-dependent thermal conductivity of CF scaffolds/PDMS composites is also investigated in Fig. 3d. As temperature raised gradually from 25 to 100 °C, thermal conductivity of all composites increased slightly and then decreased, presenting negligible change in thermal properties. The thermal conductivity of VCS-7/PDMS and HCS-7/PDMS at 100 °C can remain higher than 40 W m^−1^ K^−1^, indicating the excellent heat transfer performance in the practical device operation temperature range.

To visualize the difference in heat transfer performance in the through-plane direction caused by CF scaffolds structure, PDMS, CS-5/PDMS, DCS-10/PDMS, VCS-10/PDMS with the same thickness of 1 mm were placed on a heating plate (65 °C), and the surface temperature was monitored using an infrared thermal-imaging camera. The thermographic images and temperature curves varying with time are shown in Fig. 3e, f. A series of infrared images are exhibited after heating for 5, 10, 20, 30, 60 and 90 s, respectively. As the heating time increased, the color of infrared images became brighter gradually. The surface temperature of VCS-10/PDMS increased rapidly, which showed largest slope in temperature curves among all samples. After heating for 60 s, the surface temperature of VCS-10/PDMS raised to 62.1 °C while it was 59.5 and 60.2 °C for PDMS and CS-5/PDMS, respectively. The fast temperature rising rate and high equilibrium temperature of VCS-10/PDMS indicated that VCS can construct highly efficient heat transfer paths in the thickness direction.

In addition, to further demonstrate the efficient heat transfer of VCS in the thickness direction compared to other CF scaffolds, the transient thermal response of the different structure was simulated via finite element analysis. The simulation models are shown in Fig. 3g, and more detailed information about the parameter settings can be found in Fig. S11 and Table S3. The initial temperature of system was 25 °C and continuous temperature load of 50 °C was applied to the bottom of the models, which generated unidirectional heat conduction across the models along the through-plane direction. The temperature profiles at the top side of modules were recorded to evaluate the heat transfer capability at the same thermal response time. The simulated results at varied thermal response time are shown in Fig. 3h. It can be seen from the temperature contour that the CF formed an effective heat transfer path in PDMS, and the heat was conducted rapidly along the axial direction of fibers. Owing to the vertically arranged structure and high fiber contents, the average temperature at the top side of VCS/PDMS was 46.84 °C at 0.005 s, which was much larger than that of the CS/PDMS (25.31 °C) and HCS/PDMS (25.00 °C). Although DCS/PDMS had the same filler contents, its average temperature at the top side (36.78 °C) was lower than that of the VCS/PDMS, because the fibers were arranged diagonally and the heat transfer paths from the bottom to the top were extended. In short, simulation results demonstrated that the orderly aligned and interlinked fibers in vertical direction are crucial for VCS/PDMS in achieving outstanding thermal conductivity and directional heat transfer capability in the thickness direction. It is worth noting that the VCS-7/PDMS and DCS-7/PDMS presented high through-plane thermal conductivity compared to the reported composites with oriented or framework structure, which indicated the significant advantage of our aligned and interconnected CF scaffolds in forming efficient heat transfer paths (Fig. 3i and Table S4).

### Thermal Management Performance of CF Scaffolds/PDMS Composites

**Fig. 4:**
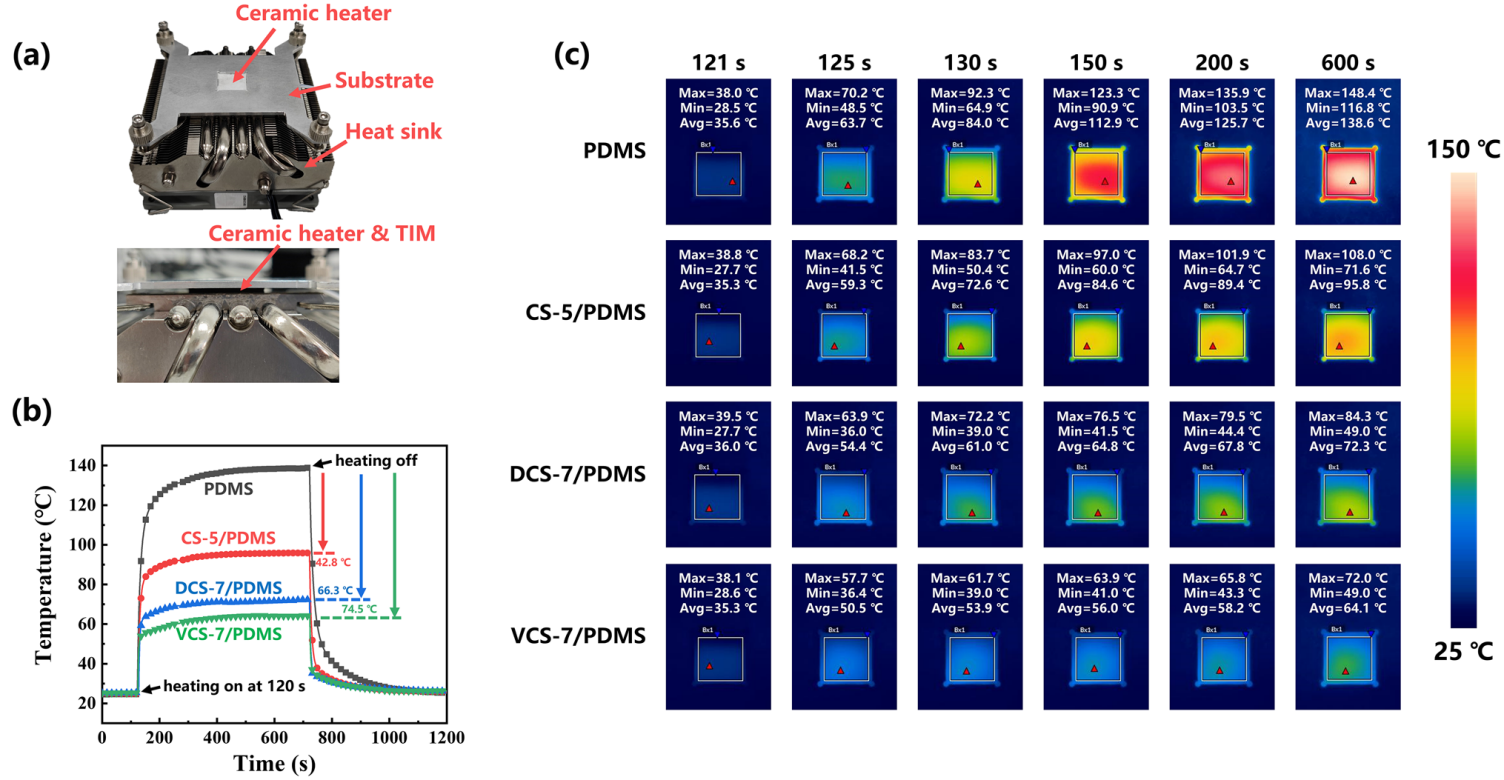
**a** Schematic configuration of the thermal management performance evaluation system. **b** Temperature variation of the ceramic heater as function of the heating time. **c** Infrared thermal photographs of surface temperature of ceramic heater at different times

To investigate and compare the cooling performance in real cases, an evaluation system consisting of ceramic heater, heat sink, TIM and substrate was built to simulate the heat dissipation process in electronic components. As seen in Fig. 4a, PDMS, SC-5/PDMS, DCS-7/PDMS and VCS-7/PDMS with the same bond line thickness of 800 μm and lateral size of *Φ*25.4 mm were placed between the ceramic heater (20 W, 16 mm × 16 mm × 2 mm) and heat sink. The heat sink was connected to a forced air-cooling system to extract the generated heat. In Fig. 4b, the heating was turned on at 120 s and then the temperature increased sharply in a short time. After the temperature reached the equilibrium, the heating was turned off at 720 s to cool down the heater. It is observed that the steady-state temperature of the heater with PDMS, SC-5/PDMS, DCS-7/PDMS and VCS-7/PDMS after heating 600 s was 138.6, 95.8, 72.3 and 64.1 °C, respectively, and the corresponding infrared thermal images are displayed in Fig. 4c. The great decrease in temperature revealed that the use of VCS-7/PDMS demonstrated greater cooling efficiency compared to others.

**Fig. 5:**
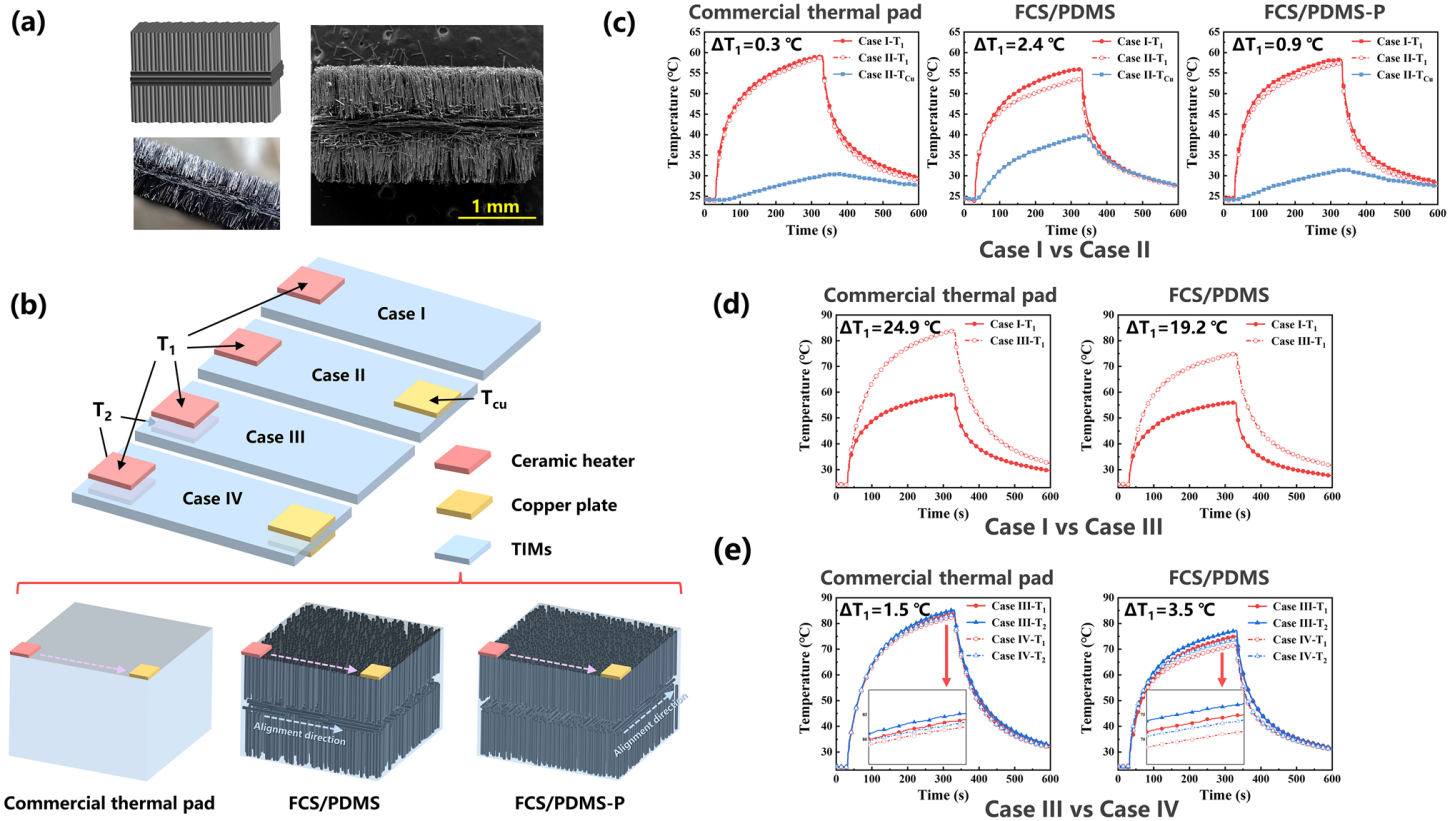
**a** Photograph and SEM image of prepared FCS. **b** Schematic of four cases designed to prove the directional heat transfer capability of FCS/PDMS. **c** Temperature evolution of ceramic heater and copper plate for commercial thermal pad, FCS/PDMS and FCS/PDMS-P in case I and case II. Temperature curves of ceramic heaters for commercial thermal pad and FCS/PDMS in **d** case I & case III and **e** case III & case IV

**Fig. 6:**
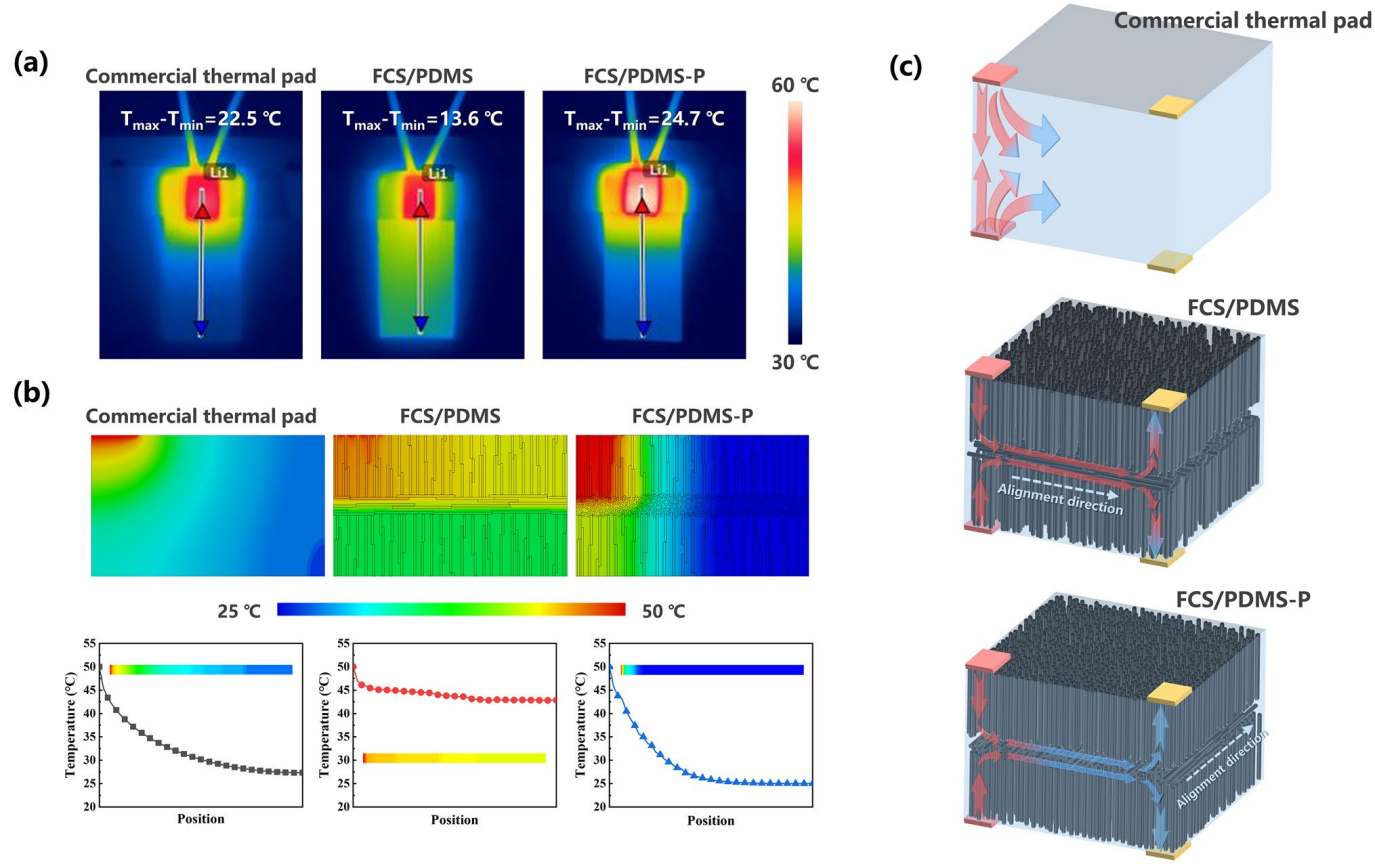
**a** Infrared thermal photographs of surface temperature of commercial thermal pad, FCS/PDMS and FCS/PDMS-P after heating 120 s. **b** Transient temperature distribution of commercial thermal pad, FCS/PDMS and FCS/PDMS-P calculated by finite element analysis. **c** Schematics of the tuned heat transfer paths for efficient heat dissipation

Considering the miniaturization of electronic devices and the complexity of electronic packaging, TIMs with tunable heat transfer path could make the design of thermal management system more flexible. The efficient heat transfer on the customized paths can be realized by combining CF with different alignment direction, since the prepared CF scaffolds with oriented structure reveal excellent directional heat transfer capability in fiber alignment direction. Here, fishbone-shaped CF scaffolds (FCS) with the combination of vertically and horizontally aligned CF was prepared by multiple stacking and carbonization process, its cross-sectional morphology can be seen in Fig. 5a. To confirm the turning of heat transfer paths, ceramic heater (0.4 W, 7 mm × 5 mm × 1.2 mm) and copper plate (10 mm × 10 mm × 1.2 mm) were used as heat source and heat sink, respectively, and four cases, as illustrated in Fig. 5b, were designed to compare the cooling effect of the FCS/PDMS, FCS/PDMS-P and the commercial thermal pad (5 W m^−1^ K^−1^) with the same size of 27 mm × 12 mm × 1.8 mm. In the cases I and II, single heat source was set on the left of the upper surface of TIMs, while an additional copper plate was provided on the right of upper surface in case II. In particular, the line connecting the heater and copper plate was parallel to the horizontally arranged fibers in FCS/PDMS, while the line was perpendicular to the horizontally arranged fibers in FCS/PDMS-P. The temperature of ceramic heaters (*T*_1_ and *T*_2_) and copper plate (*T*_cu_) was measured by using a thermocouple. From the recorded temperature curves (Fig. 5c), it is observed that the *T*_Cu_ for FCS/PDMS shows faster raising rate and higher value than that of the commercial thermal pad and FCS/PDMS-P. And the *T*_1_ difference for FCS/PDMS between case I and case II reaches 2.4 °C, which is much higher than that for commercial thermal pad (0.3 °C) and FCS/PDMS-P (0.9 °C), indicating that FCS/PDMS shows better cooling effect due to the efficient heat transfer along the tuned paths. As for the two heat sources arranged on the upper and lower surface in case III, the temperature of heat source increased distinctly because of the inevitable thermal interference. However, compared to commercial thermal pad, FCS/PDMS shows smaller increment of the *T*_1_ between case I and case III (Fig. 5d). It is believed that the horizontally aligned fibers in the middle of FCS/PDMS have small thermal conductivity and tend to transfer the heat in a horizontal direction, which can weaken the thermal interference between the top and bottom heat sources. Moreover, after setting the copper plates in case IV, the FCS/PDMS also exhibits superior cooling effect with obvious drop in temperature compared to commercial thermal pad (Fig. 5e). For case I, infrared thermal-imaging camera were carried out to visualize the variation of surface temperature of TIMs. As the ceramic heater was fixed at one end of TIMs, the temperature of the other end will gradually increase with heating time. After heating 120 s, the surface temperature of FCS/PDMS increased obviously and the temperature difference between the heater and the other end was 13.6 °C. While the temperature at the other end of commercial thermal pad and FCS/PDMS-P were comparable to the ambient temperature, with the temperature differences of 22.5 and 24.7 °C, respectively (Fig. 6a). In addition, as shown in Figs. 6b and S12, finite element analysis was used to simulate the process, and the tendency of temperature change in the simulation results was consistent with the actual measurement. Thus, through the combination of fibers with vertical orientation and horizontal orientation, FCS/PDMS can transfer the heat effectively to the copper plate placed in the same plane as the ceramic heater along the customized transfer paths (Fig. 6c). The customized heat transfer path could also mitigate the thermal interference between heat sources to a certain extent, allowing different heat sources to share the same TIM, providing versatility for the design of thermal management systems.

## Conclusions

In summary, toward fully utilizing the excellent axial thermal conductivity of CF for efficient heat dissipation, we tailored a highly ordered CF scaffold in PDMS matrix with varied alignment direction. By regulating the magnetic direction and initial stacking density, three CF scaffolds with HCS, DCS and VCS fibers were developed via magnetic field-assisted Tetris-style stacking and carbonization process. As a result, the resultant HCS-7/PDMS and VCS-7/PDMS exhibited a high thermal conductivity of 42.18 and 45.01 W m^−1^ K^−1^ in fiber alignment direction, respectively, dramatically enhancing the thermal conductivity of PDMS by about 209 and 224 times. In addition, due to the vertically aligned CF forming effective phonon transport pathway in the through-plane direction, the VCS-7/PDMS as a TIM for cooling the ceramic heater showed the most significant reduction in temperature compared to that of other samples. Furthermore, combining CF with different alignment direction could realize the regulation of the heat transfer paths, since the thermal conductivity of prepared CF scaffolds with oriented structure revealed high anisotropy ratio. Fishbone-shaped CF scaffold was successfully prepared by multiple stacking and carbonization process, and the obtained FCS/PDMS can effectively direct heat along customized paths, which demonstrated more excellent cooling effect compared to commercial thermal pad in special scenarios. This work provides insights into the construction of CF-based thermally conductive composites, and the proposed strategy is relatively more flexible and cost-effective compared to the technique ever reported. The tailored CF scaffolds with varied alignment direction could customize the heat transfer paths, which diversifies the design of thermal management system.

### Supplementary Information

Below is the link to the electronic supplementary material.

Supplementary file1 (MP4 210223 KB)

Supplementary file2 (PDF 1414 KB)
