# Supplementary material for: Tetris-Style Stacking Process to Tailor the Orientation of Carbon Fiber Scaffolds for Efficient Heat Dissipation
Source: Nanomicro Lett. 2023 Jun 7;15:146. doi: 10.1007/s40820-023-01119-0 (PMC10247643; doi:10.1007/s40820-023-01119-0)
Supplement: Supplementary file 2 — Supplementary file2 (PDF 1414 KB) [file 40820_2023_1119_MOESM2_ESM.pdf]

Supporting Information for

Tetris-Style Stacking Process to Tailor the Orientation of Carbon Fiber Scaffolds for Efficient Heat Dissipation

Shida Han[1], Yuan Ji[1], Qi Zhang[1], Hong Wu[1, *], Shaoyun Guo[1, *], Jianhui Qiu[2], Fengshun Zhang[3]

[1]The State Key Laboratory of Polymer Materials Engineering, Sichuan Provincial Engineering Laboratory of Plastic/Rubber Complex Processing Technology, Polymer Research Institute of Sichuan University, Chengdu 610065, P. R. China

[2]Department of Mechanical Engineering, Faculty of Systems Science and Technology, Akita Prefectural University, Akita 015-0055, Japan

[3]Institute of Chemical Materials, China Academy of Engineering Physics, Mianyang 621900, P. R. China

*Corresponding authors. E-mail: wh@scu.edu.cn (Hong Wu), nic7702@scu.edu.cn (Shaoyun Guo)

Supplementary Figures and Tables

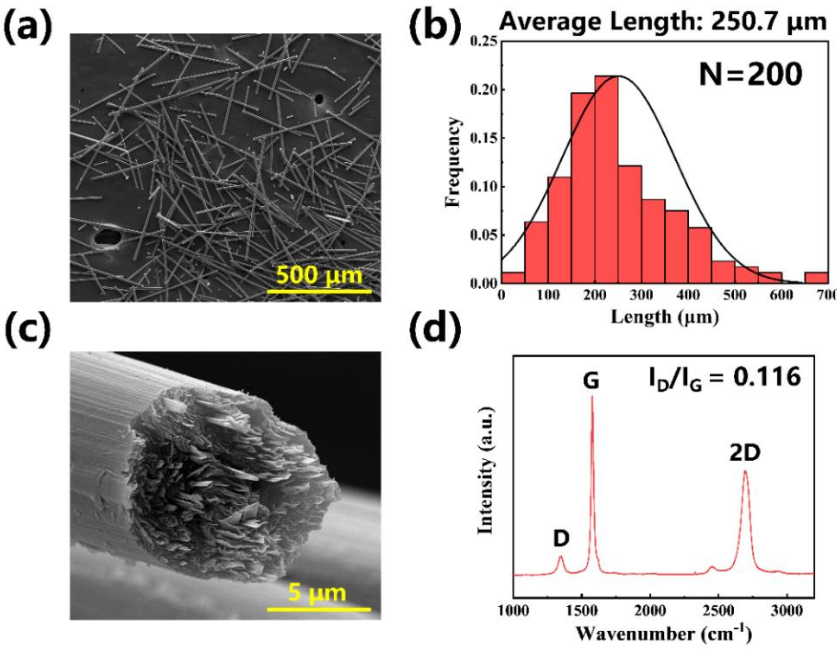

Fig. S1 (**a**) SEM image of Pitch-based CF used in this work and (**b**) the statistics of fiber length, showing an average length of 250.7 μm. (**c**) Highly ordered graphite structure and (**d**) Raman spectrum of Pitch-based CF indicates excellent axial thermal conductivity

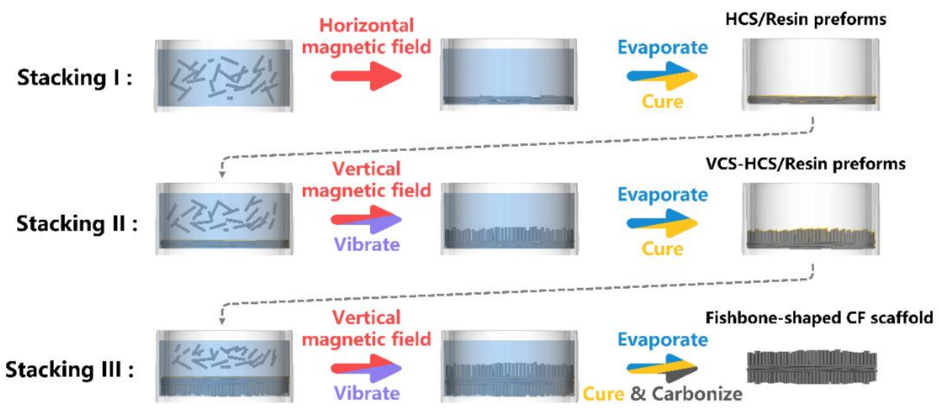

Fig. S2 Schematics of preparation of Fishbone-shaped CF scaffolds (FCS) via multiple stacking and carbonization process

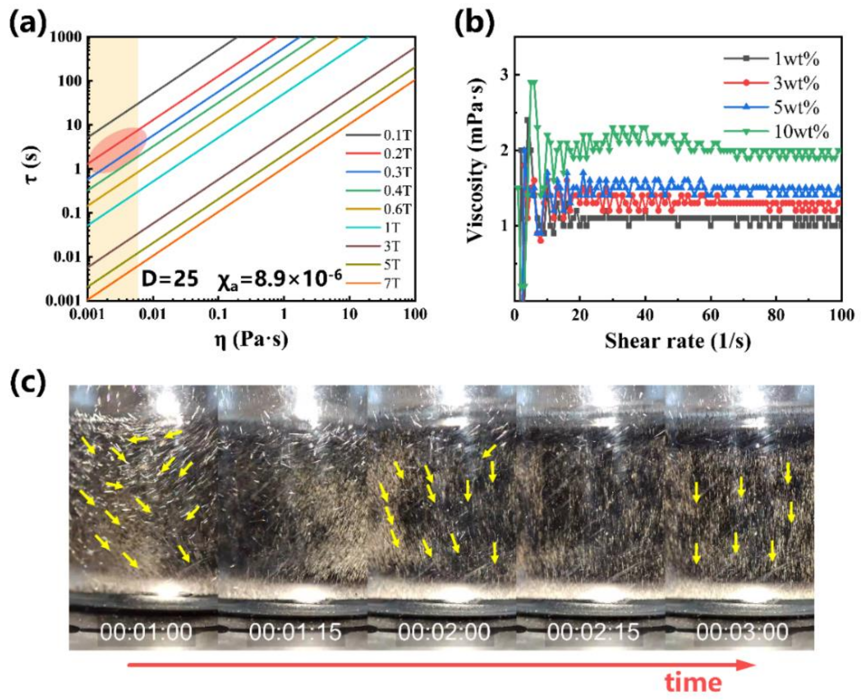

Fig. S3 (**a**) The variation curve of alignment relaxation time (τ) versus the viscosity (η) of solution under different magnetic field strength. (**b**) Apparent viscosity of phenolic resin/ethanol solutions at different concentrations. (**c**) Images of fiber deposition process in ethanol under the vertical magnetic field and CF completed the orientation in about 2 s before landing.

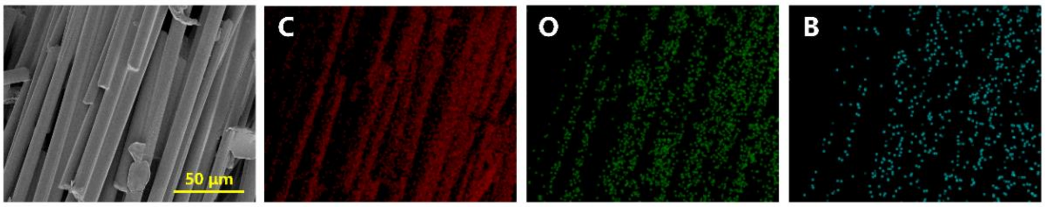

Fig. S4 Cross-sectional SEM image of CF/resin preforms and corresponding EDS mappings of C, O and B elemental distributions

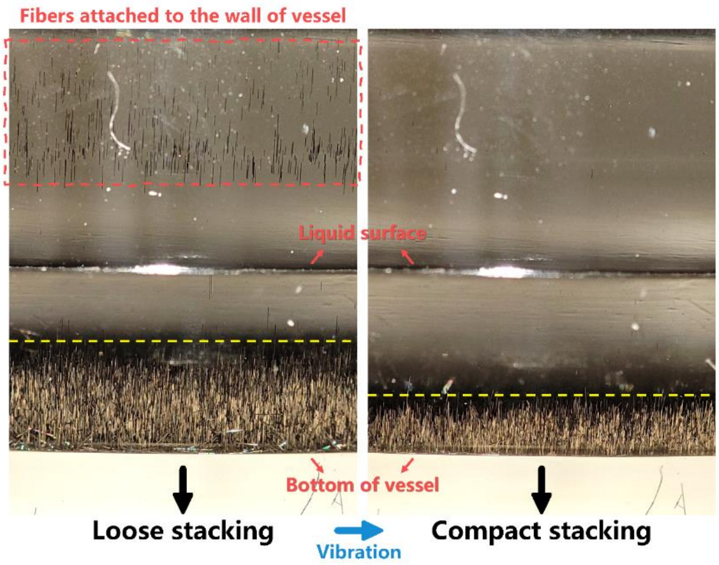

Fig. S5 The height of the stacked fibers was apparently decreased after vibration, showing compact structure with enhanced initial stacking density

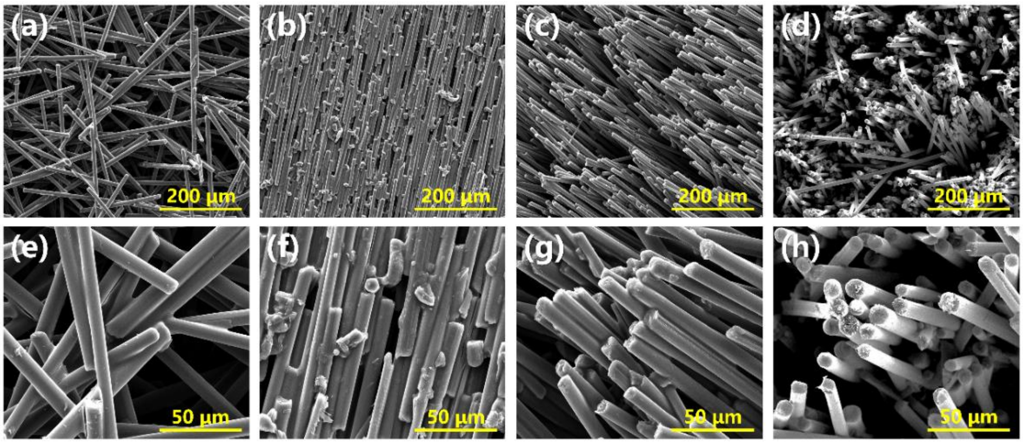

Fig. S6 Local surface morphology of (**a, e**) CS-5, (**b, f**) HCS-7, (**c, g**) DCS-7 and (**d, h**) VCS-7

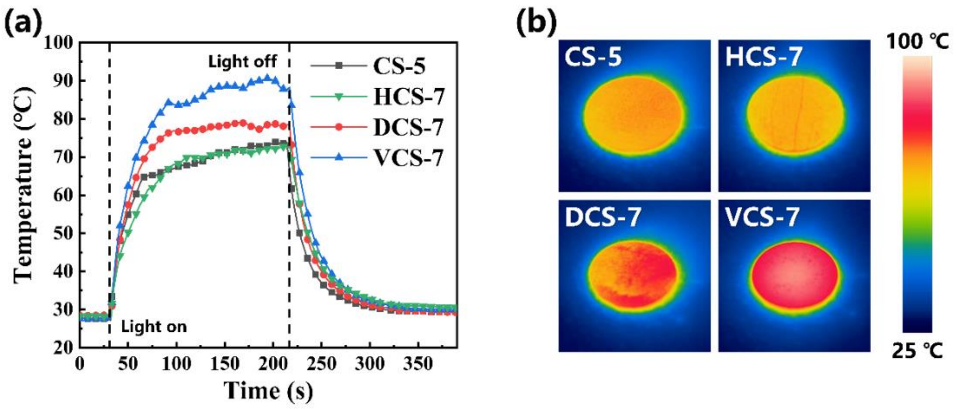

Fig. S7 (**a**) Temperature curves of CF scaffolds surface varied with time under the light density of 100 mW cm^{-2} and corresponding infrared thermal images at 200 s were illustrated in (**b**)

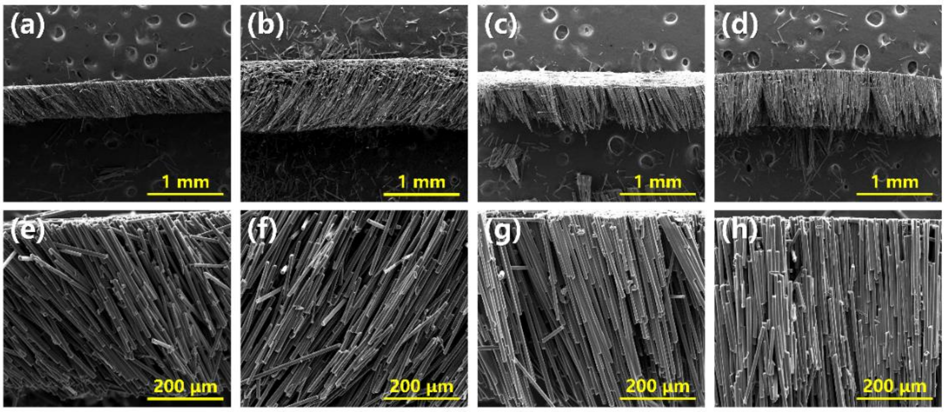

Fig. S8 Cross-sectional SEM images of CF scaffolds: (**a, e**) DCS-5, (**d, f**) DCS-10, (**c, g**) VCS-5 and (**d, h**) VCS-10

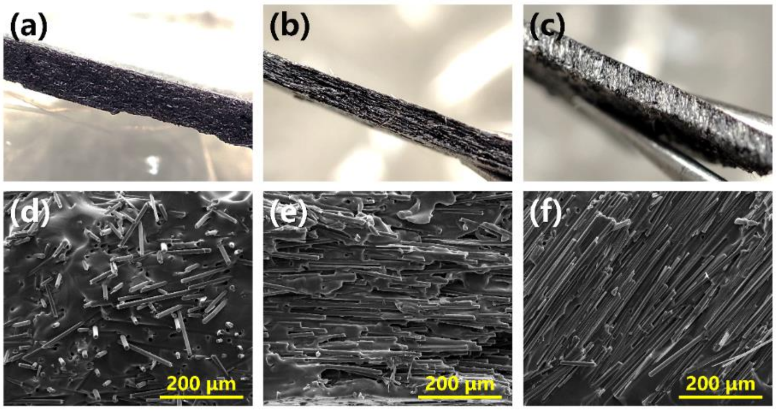

Fig. S9 Macro photos and SEM images of (**a, d**) CS-5/PDMS, (**b, e**) HCS-7/PDMS and (**c, f**) DCS-7/PDMS prepared by impregnation of PDMS into CF scaffolds

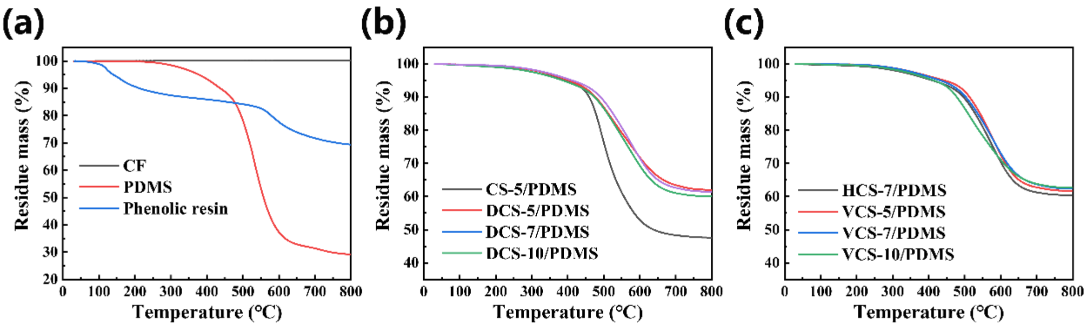

Fig. S10 TGA curves of (**a**) neat PDMS, CF and (**b**) CF scaffolds/PDMS composites in the nitrogen atmosphere. The mass fraction of CF scaffolds (M_{CF}) was calculated using the equation of $M_{CF} = \frac{W_{Compsite} - W_{PDMS}}{1 - W_{PDMS}}$, where W_{PDMS} and $W_{Composites}$ are the residue mass of PDMS and CF scaffolds/PDMS at 800 °C, respectively. And the volume fraction of CF scaffolds (V_{CF}) can be determined by the formula of $V_{CF} = \frac{M_{CF}/\rho_{CF}}{1/\rho_{Composites}}$, where ρ_{CF} and $\rho_{Composites}$ are density of CF (2.22 g cm^{-3}) and composites. The calculated results can be seen in Table S2

Table S1 The calculated mass fraction and volume fraction of fillers in the CF scaffolds/PDMS

	**Residue mass at 800 °C (%)**	M_{CF} **(wt%)**	V_{CF} **(vol%)**
PDMS	29.16	-	-
CS-5/PDMS	47.6	26.0	14.3
DCS-5/PDMS	61.99	46.3	28.3
DCS-7/PDMS	61.45	45.6	27.7
DCS-10/PDMS	60.05	43.6	25.9
VCS-5/PDMS	61.72	46.0	28.0
VCS-7/PDMS	62.38	46.9	29.0
VCS-10/PDMS	62.69	47.3	29.7
HCS-7/PDMS	60.39	44.1	26.6

Table S2 The parameters for calculating thermal conductivity of CF scaffolds/PDMS composites. The specific heat capacity (C_p) of all samples was evaluated using a differential scanning calorimeter (DSC) analysis. The density (ρ) of all samples was obtained by the water displacement method

	**Thermal diffusivity (mm^2 s^{-1})**	**Specific heat capability (J g^{-1} °C^{-1})**	**Density (g cm^{-3})**	**Thermal conductivity (W m^{-1} K^{-1})**	**Direction**
CS-5/PDMS	5.81 ± 0.36<br>1.48 ± 0.13	1.205	1.220	8.54 ± 0.53<br>2.18 ± 0.19	$k_{In\text{-}plane}$<br>$k_{Through\text{-}plane}$
DCS-5/PDMS	7.50 ± 0.46<br>23.57 ± 1.72	1.106	1.355	11.24 ± 0.69<br>35.32 ± 2.58	$k_{In\text{-}plane}$<br>$k_{Through\text{-}plane}$
DCS-7/PDMS	4.46 ± 0.29<br>24.20 ± 1.98	1.160	1.347	6.97 ± 0.45<br>37.81 ± 3.09	$k_{In\text{-}plane}$<br>$k_{Through\text{-}plane}$
DCS-10/PDMS	7.28 ± 0.53<br>14.52 ± 1.20	1.108	1.317	10.62 ± 0.77<br>21.19 ± 1.75	$k_{In\text{-}plane}$<br>$k_{Through\text{-}plane}$
VCS-5/PDMS	2.29 ± 0.16<br>25.46 ± 1.87	1.147	1.352	3.55 ± 0.25<br>39.48 ± 2.90	$k_{In\text{-}plane}$<br>$k_{Through\text{-}plane}$
VCS-7/PDMS	2.39 ± 0.23<br>27.60 ± 1.84	1.188	1.373	3.90 ± 0.38<br>45.01 ± 3.00	$k_{In\text{-}plane}$<br>$k_{Through\text{-}plane}$
VCS-10/PDMS	3.07 ± 0.35<br>27.02 ± 2.11	1.093	1.394	4.68 ± 0.53<br>41.16 ± 3.21	$k_{In\text{-}plane}$<br>$k_{Through\text{-}plane}$
HCS-7/PDMS	1.56 ± 0.18<br>29.64 ± 1.93	1.062	1.340	2.21 ± 0.26<br>42.18 ± 2.75	$k_{\perp}$<br>$k_{//}$

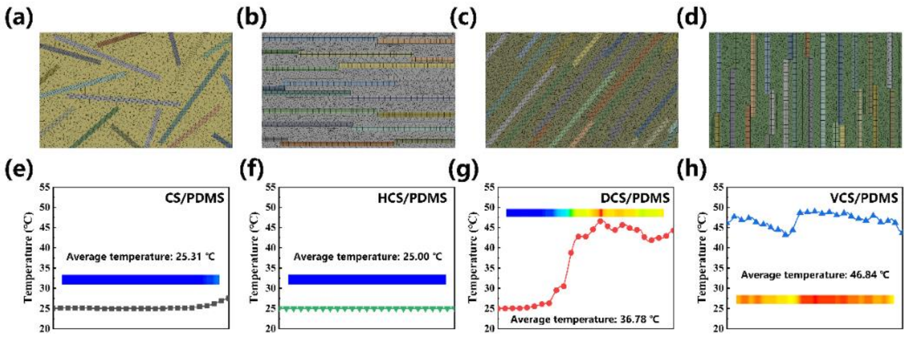

Fig. S11 Schematic of ANSYS simulation models with grid division of (**a**) CS/PDMS, (**b**) HCS/PDMS (**c**) DCS/PDMS and (**d**) VCS/PDMS. (**e-h**) The top side temperature profiles and corresponding simulation boxes of different models

Finite element simulation was performed to analyze the transient thermal response and heat transfer capability of the carbon scaffolds/PDMS composites with different structure. As shown in Fig. S11(a-d), the computation domain is set to 0.42 × 0.252 mm^2 and the fiber region is introduced based on the structure and volume fraction of each composite. Major parameters of PDMS and CF in the simulation analysis can be found in Table S3.

In a general simulation, a linear heat source with temperature of 50 ℃ is set at the bottom of the models and the convection coefficient of the top side was set as 10 W m^{-2} K^{-1} with the ambient temperature of 25 ℃. Fig. S11(d-f) show the temperature profiles at the top side of different models, and the results demonstrate that the VCS/PDMS have a higher temperature compared to composites with other CF scaffolds, indicating the excellent heat transfer ability in through-plane direction.

Table S3 Parameters of PDMS and CF used in the simulation analysis

**Parameters**	**PDMS**	**CF**	
Thermal conductivity ($W\ m^{-1}\ K^{-1}$)	0.2	Axial direction	Radial direction
		900	10
Specific heat capability ($J\ g^{-1}$ ℃$^{-1}$)	1.42	0.71	
Density ($g\ cm^{-3}$)	1.04	2.22	

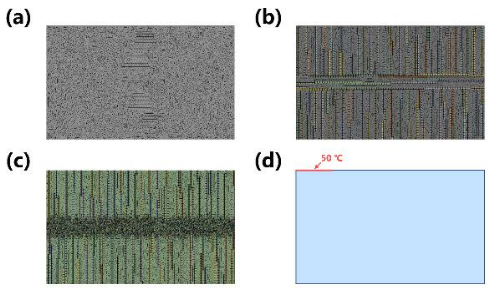

Fig. S12 Schematic of ANSYS simulation models with grid division of (**a**) commercial thermal pad, (**b**) FCS/PDMS and (**c**) FCS/PDMS-P. The main heat source (50 ℃) was set at the upper left end of the models (**d**)

Table S4 Comparison of thermal conductivities of our CF scaffolds/PDMS with reported thermally conductive composites [S1-S23]

Filler	Matrix	Filler contents (vol%)	Thermal conductivity (W m^{-1} K^{-1})	Direction	Refs.
**Carbon fiber-based thermally conductive composites**					
CF	Fluorinated rubber	11.5	23.3	$k_{Through\text{-}plane}$	S1
CF	Olefin block copolymer	30	15.06	$k_{Through\text{-}plane}$	S2
CF	PDMS	27.3	34.94	$k_{Through\text{-}plane}$	S3
CF	PDMS	20	11.76	$k_{Through\text{-}plane}$	S4
CF	Epoxy resin	31	32.6	$k_{Through\text{-}plane}$	S5
CF	PDMS	9	4.72	$k_{Through\text{-}plane}$	S6
CF	PDMS	12.8	6	$k_{Through\text{-}plane}$	S7
CF	PDMS	20	43.47	$k_{Through\text{-}plane}$	S8
CF/Al_2O_3	PDMS	24/47	38	$k_{Through\text{-}plane}$	S9
CF/Al_2O_3	PDMS	20/50	26.49	$k_{Through\text{-}plane}$	S10
Ni@CF	PDMS	21.5	10.5	$k_{Through\text{-}plane}$	S11
CF	PDMS	31.49	35.5	$k_{In\text{-}plane}$	S12
**Graphite monolith-based thermally conductive composites**					
Graphite frameworks	PDMS	17.6	35.4	$k_{Through\text{-}plane}$	S13
Graphite aerogel	Epoxy resin	0.91	4.14	$k_{Through\text{-}plane}$	S14
Graphite aerogel	PDMS	5.89	9.92	$k_{Through\text{-}plane}$	S15
Graphite fiber monolith	Paraffin wax	20.7	36.49	$k_{In\text{-}plane}$	S16
**Ceramic monolith-based thermally conductive composites**					
SiC frameworks	Epoxy resin	21	10.27	$k_{Through\text{-}plane}$	S17
3D aligned BA	Epoxy resin	40	21	$k_{Through\text{-}plane}$	S18
LM/BN	Polyethylene glycol	30	8.8	$k_{Through\text{-}plane}$	S19
3D BN foam	Epoxy resin	59.43	6.11	$k_{Through\text{-}plane}$	S20
AlN honeycomb	Epoxy resin	47.26	9.48	$k_{Through\text{-}plane}$	S21
BN-SiC skeleton	PDMS	8.35	3.87	$k_{Through\text{-}plane}$	S22
BN	PVP	62	12.1	$k_{Through\text{-}plane}$	S23
**This work**					
HCS-7	PDMS	26.6	42.18	$k_{//}$	
DCS-7	PDMS	27.7	37.8	$k_{Through\text{-}plane}$	
VCS-7	PDMS	29	45.01	$k_{Through\text{-}plane}$	

Supplementary References

[S1] K. Uetani, S. Ata, S. Tomonoh, T. Yamada, M. Yumura et al., Elastomeric thermal interface materials with high through-plane thermal conductivity from carbon fiber fillers vertically aligned by electrostatic flocking. Adv. Mater. **26**(33), 5857-5862 (2014). https://doi.org/10.1002/adma.201401736

[S2] G. Zhang, S. Xue, F. Chen, Q. Fu, An efficient thermal interface material with anisotropy orientation and high through-plane thermal conductivity. Compos. Sci. Technol. **231**, 109784 (2023). https://doi.org/10.1016/j.compscitech.2022.109784

[S3] Z. Zhang, J. Wang, J. Shang, Y. Xu, Y.J. Wan et al., A through-thickness arrayed carbon fibers elastomer with horizontal segregated magnetic network for highly efficient thermal management and electromagnetic wave absorption. Small **19**(4), e2205716 (2022). https://doi.org/10.1002/smll.202205716

[S4] Q. Wu, J. Miao, W. Li, Q. Yang, Y. Huang et al., High-performance thermal interface materials with magnetic aligned carbon fibers. Materials **15**(3), 735 (2022). https://doi.org/10.3390/ma15030735

[S5] M.H. Li, Z. Ali, X.Z. Wei, L.H. Li, G.C. Song et al., Stress induced carbon fiber orientation for enhanced thermal conductivity of epoxy composites. Compos. Part B. Eng. **208**, 108599 (2021). https://doi.org/10.1016/j.compositesb.2020.108599

[S6] D.L. Ding, R.Y. Huang, X. Wang, S.Y. Zhang, Y. Wu et al., Thermally conductive silicone rubber composites with vertically oriented carbon fibers: A new perspective on the heat conduction mechanism. Chem. Eng. J. **441**, 136104 (2022). https://doi.org/10.1016/j.cej.2022.136104

[S7] X. Hou, Y.P. Chen, W. Dai, Z.W. Wang, H. Li et al., Highly thermal conductive polymer composites via constructing micro-phragmites communis structured carbon fibers. Chem. Eng. J. **375**, 121921 (2019). https://doi.org/10.1016/j.cej.2019.121921

[S8] J. Li, Z. Ye, P. Mo, Y. Pang, E. Gao et al., Compliance-tunable thermal interface materials based on vertically oriented carbon fiber arrays for high-performance thermal management. Compos. Sci. Technol. **234**, 109948 (2023). https://doi.org/10.1016/j.compscitech.2023.109948

[S9] R.Y. Huang, D.L. Ding, X.X. Guo, C.J. Liu, X.H. Li et al., Improving through-plane thermal conductivity of pdms-based composites using highly oriented carbon fibers bridged by al2o3 particles. Compos. Sci. Technol. **230**, 109717 (2022). https://doi.org/10.1016/j.compscitech.2022.109717

[S10] Q. Wu, W.J. Li, C. Liu, Y.W. Xu, G.G. Li et al., Carbon fiber reinforced elastomeric thermal interface materials for spacecraft. Carbon **187**, 432-438 (2022). https://doi.org/10.1016/j.carbon.2021.11.039

[S11] X. Zhang, S. Zhou, B. Xie, W. Lan, Y. Fan et al., Thermal interface materials with sufficiently vertically aligned and interconnected nickel-coated carbon fibers under high filling loads made via preset-magnetic-field method. Compos. Sci. Technol. **213**, 108922 (2021). https://doi.org/10.1016/j.compscitech.2021.108922

[S12] X.F. Zhang, B. Xie, S.L. Zhou, X. Yang, Y.W. Fan et al., Radially oriented functional thermal materials prepared by flow field-driven self-assembly strategy. Nano Energy **104**, 107986 (2022). https://doi.org/10.1016/j.nanoen.2022.107986

[S13] F. Zhang, D.H. Ren, Y.H. Zhang, L.Q. Huang, Y.X. Sun et al., Production of highly-oriented graphite monoliths with high thermal conductivity. Chem. Eng. J.

431, 134102 (2022). https://doi.org/10.1016/j.cej.2021.134102

[S14] M. Li, J. Liu, S. Pan, J. Zhang, Y. Liu et al., Highly oriented graphite aerogel fabricated by confined liquid-phase expansion for anisotropically thermally conductive epoxy composites. ACS Appl. Mater. Interfaces **12**(24), 27476-27484 (2020). https://doi.org/10.1021/acsami.0c02151

[S15] J.X. Zeng, Z.Q. Chen, M.X. Li, Y.X. Guo, J. Xu et al., Carbon aerogel with high thermal conductivity enabled by shrinkage control. Chem. Mater. **34**(20), 9172-9181 (2022). https://doi.org/10.1021/acs.chemmater.2c02133

[S16] Z. Jiang, T. Ouyang, L. Ding, W. Li, W.W. Li et al., 3D self-bonded porous graphite fiber monolith for phase change material composite with high thermal conductivity. Chem. Eng. J. **438**, 135496 (2022). https://doi.org/10.1016/j.cej.2022.135496

[S17] X.N. Zhou, S.S. Xu, Z.Y. Wang, L.C. Hao, Z.Q. Shi et al., Wood-derived, vertically aligned, and densely interconnected 3d sic frameworks for anisotropically highly thermoconductive polymer composites. Adv. Sci. **9**(7), e2103592 (2022). https://doi.org/10.1002/advs.202103592

[S18] Y. Cui, Z. Qin, H. Wu, M. Li, Y. Hu, Flexible thermal interface based on self-assembled boron arsenide for high-performance thermal management. Nat. Commun. **12**(1), 1284 (2021). https://doi.org/10.1038/s41467-021-21531-7

[S19] C. Guo, L. He, Y. Yao, W. Lin, Y. Zhang et al., Bifunctional liquid metals allow electrical insulating phase change materials to dual-mode thermal manage the li-ion batteries. Nano-Micro Lett. **14**(1), 202 (2022). https://doi.org/10.1007/s40820-022-00947-w

[S20] X.W. Xu, R.C. Hu, M.Y. Chen, J.F. Dong, B. Xiao et al., 3D boron nitride foam filled epoxy composites with significantly enhanced thermal conductivity by a facial and scalable approach. Chem. Eng. J. **397**, 125447 (2020). https://doi.org/10.1016/j.cej.2020.125447

[S21] Z.L. Wei, W.Q. Xie, B.Z. Ge, Z.J. Zhang, W.L. Yang et al., Enhanced thermal conductivity of epoxy composites by constructing aluminum nitride honeycomb reinforcements. Compos. Sci. Technol. **199**, 108304 (2020). https://doi.org/10.1016/j.compscitech.2020.108304

[S22] Y. Yao, Z. Ye, F. Huang, X. Zeng, T. Zhang et al., Achieving significant thermal conductivity enhancement via an ice-templated and sintered bn-sic skeleton. ACS Appl. Mater. Interfaces **12**(2), 2892-2902 (2020). https://doi.org/10.1021/acsami.9b19280

[S23] H. He, W. Peng, J. Liu, X.Y. Chan, S. Liu et al., Microstructured bn composites with internally designed high thermal conductivity paths for 3d electronic packaging. Adv. Mater. **34**(38), e2205120 (2022). https://doi.org/10.1002/adma.202205120
